# Risk factors associated with bacteremia in COVID-19 patients admitted to intensive care unit: a retrospective multicenter cohort study

**DOI:** 10.1007/s15010-022-01853-4

**Published:** 2022-06-10

**Authors:** Cecilia Bonazzetti, Matteo Rinaldi, Andrea Giacomelli, Riccardo Colombo, Davide Ottolina, Sara Giordana Rimoldi, Cristina Pagani, Valentina Morena, Anna Lisa Ridolfo, Oana Vatamanu, Maria Eugenia Giacomini, Caterina Campoli, Letizia Oreni, Giuliano Rizzardini, Pierluigi Viale, Spinello Antinori, Maddalena Giannella

**Affiliations:** 1 Department of Infectious Diseases, ASST Fatebenefratelli-Sacco, Luigi Sacco University Hospital, Milan, Italy; 2 Luigi Sacco Department of Biomedical and Clinical Sciences, Università degli Studi di Milano, III Infectious Diseases Unit, Luigi Sacco Hospital, Via G.B. Grassi 74, 20157 Milan, Italy; 3 Infectious Diseases Unit IRCCS, Policlinico Sant’Orsola, Department Medical Surgical Science, University of Bologna, Bologna, Italy; 4 Department of Anesthesiology and Intensive Care, ASST Fatebenefratelli-Sacco, Luigi Sacco Hospital, Milan, Italy; 5 Clinical Microbiology, Virology and Bioemergency, ASST Fatebenefratelli Sacco, Luigi Sacco Hospital, Milan, Italy

**Keywords:** Bacteremia, COVID-19, SOFA score, Charlson score, Immunosuppressive therapy

## Abstract

**Purpose:**

This multicenter observational study was done to evaluate risk factors related to the development of BSI in patients admitted to ICU for COVID-19.

**Methods:**

All patients with COVID-19 admitted in two COVID-19 dedicated ICUs in two different hospital between 02–2020 and 02–2021 were recruited.

**Result:**

537 patients were included of whom 265 (49.3%) experienced at least one BSI. Patients who developed bacteremia had a higher SOFA score [10 (8–12) vs 9 (7–10), *p* < 0.001], had been intubated more frequently [95.8% vs 75%, *p* < 0.001] and for a median longer time [16 days (9–25) vs 8 days (5–14), *p* < 0.001]. Patients with BSI had a median longer ICU stay [18 days (12–31.5) vs 9 days (5–15), *p* < 0.001] and higher mortality [54% vs 42.3%, *p* < 0.001] than those who did not develop it. Development of BSI resulted in a higher SOFA score [aHR 1.08 (95% CI 1.03–1.12)] and a higher Charlson score [csAHR 1.15 (95% CI 1.05–1.25)].

**Conclusion:**

A high SOFA score and a high Charlson score resulted associated with BSI’s development. Conversely, immunosuppressive therapy like steroids and tocilizumab, has no role in increasing the risk of bacteremia.

**Supplementary Information:**

The online version contains supplementary material available at 10.1007/s15010-022-01853-4.

## Introduction

Severe acute respiratory syndrome coronavirus-2 (SARS-CoV-2) has caused one of the largest pandemics that health systems have faced. Although the clinical course of most cases is mild, 15% of patients develop severe disease [1] and require admission to intensive care unit (ICU) and ventilatory support due to respiratory failure [1, 2].

The modifications in hospital organization in terms of infrastructures and human resources, the use of immunosuppressive drugs for the treatment of COVID-19 patients, and the pathogenetic mechanism of SARS-CoV-2, predisposed COVID-19 patients to health-care related infections [2]. Indeed, many studies, including systematic review and meta-analysis [3, 4] showed a marked increase in bacterial and fungal super-infections, reported in up to 24% of hospitalized COVID-19 patients [3, 5, 6]. The rate of bloodstream infections (BSIs) was higher in COVID-19 patients compared to patients not infected [7] and BSI development was associated with worse outcomes.

Little is known regarding risk factors for BSI occurrence in COVID-19 critically ill patients. Contrasting evidence of an increased risk for BSI occurrence in those exposed to immune-suppressive agents has been reported so far [8–10].

Our aim was to assess risk factors for development of BSI in a large cohort of COVID-19 patients admitted to the ICU over two pandemic waves in Northern Italy. In particular the impact of immunosuppressive therapy on the BSI risk was assessed.

## Materials and methods

### Study design and setting

This retrospective multicentric observational study was carried out in the ICU dedicated to COVID-19 patients at Luigi Sacco Hospital (tertiary 507-bed teaching), Milan and in the ICU dedicated to COVID-19 patients at Sant’Orsola-Malpighi hospital, a tertiary 1420-bed teaching hospital in Bologna.

During the study period, COVID-19 patients were managed according to clinical judgment and local policies. During the study period, there was not a predefined protocol for the management of antibiotic treatment in patients hospitalized with COVID-19 suspected of having bacterial superinfection at both centers.

### Participants

All adult (≥ 18 years) patients with COVID-19 admitted to the ICU for ≥ 48 h between 21 February 2020 and 10 February 2021.

### Variables and definitions

The primary endpoint was to evaluate the incidence of BSI episodes during ICU admission for COVID-19. BSI was defined following the Center for Disease and Control (CDC) criteria [11]. The isolation of a common skin organism usually associated with contamination had to be corroborated by two sets of blood cultures to be considered a BSI [12]. ICU-acquired bacteremia was defined as a BSI if it was diagnosed ≥ 48 h after ICU admission. To be considered a new episode a BSI had to meet the criteria for an ICU-acquired BSI due to a different organism 48 h after the initial infection. The EUCAST Expert Rules were used to define an isolated pathogen multi-drug resistant (MDR)[13].

Prior antibiotic exposure was defined as the administration of at least one antibiotic within 30 days from ICU admission. All the exposure variables were collected at the time of ICU admission and included: demographic (age, sex), obesity defined as body mass index ≥ 25, underlying diseases according to Charlson index, clinical severity evaluated through SOFA score, COVID-19 clinical course including duration of symptoms before ICU admission, length of hospitalization before ICU admission, need of mechanical ventilation, and COVID-19 treatment including remdesivir, steroids and tocilizumab. Data sources were clinical charts and hospital electronic records, collected anonymously using a predefined case report form. A careful revision of integrity and accuracy of collected data was done by a senior ID trainee (CB) before they were registered in the database.

### Statistical analysis

Demographic and clinical data are recorded as absolute numbers, proportions, percentages, and median values with their interquartile range (IQR). The clinical features of patients who developed one or more BSI were compared with those of patients without a BSI using the χ2 test (or Fisher's exact test where necessary) for categorical variables, and Wilcoxon’s rank-sum test for continuous variables.

The cumulative incidence of first BSI episodes in ICU over time was estimated comparing death and discharge from ICU as competing events. The crude incidence rate was estimated as the incidence of BSI in ICU divided by patient-days at risk with corresponding 95% confidence interval (CI), computed using a Poisson distribution.

The association of clinical and demographic variables, and BSI development was initially tested in a univariable Cox regression model to estimate the unadjusted cause-specific hazard ratio (csHR). All variables deemed to be potentially associated with the likelihood of a BSI episode in the examined ICU centers (Bologna vs Milan) such as wave, sex, age, obesity, days from symptoms to ICU’s admission, Charlson and SOFA Score, therapy with tocilizumab, remdesivir, steroids, oro-tracheal intubation (IOT) while considering tocilizumab and IOT as time-dependent variables, were included in a multivariable Cox regression model for calculating the adjusted csHR for the development of BSI, after verifying for collinearity. We verified multicollinearity among the explanatory variables by performing a preliminary check with linear regression models and evaluating the variance inflation factors (VIF). The centers were grouped for the analysis in two dummy variables according to the site location: Milan and Bologna. Since the two centers involved in the study have intrinsic different clinical and demographic characteristics, a single center-restricted analysis was also carried out to assess the consistency between the variables associated with the event in each center.

### Ethics statement

The study was approved by the *Comitato Etico Interaziendale Area 1, Milan and Comitato Etico Indipendente di Area Vasta Emilia Centro, n. 283/2020/Oss/AOUBo.* Informed consent was waived due to the study design.

## Results

### Incidence and isolate’s characteristics

**Fig. 1:**
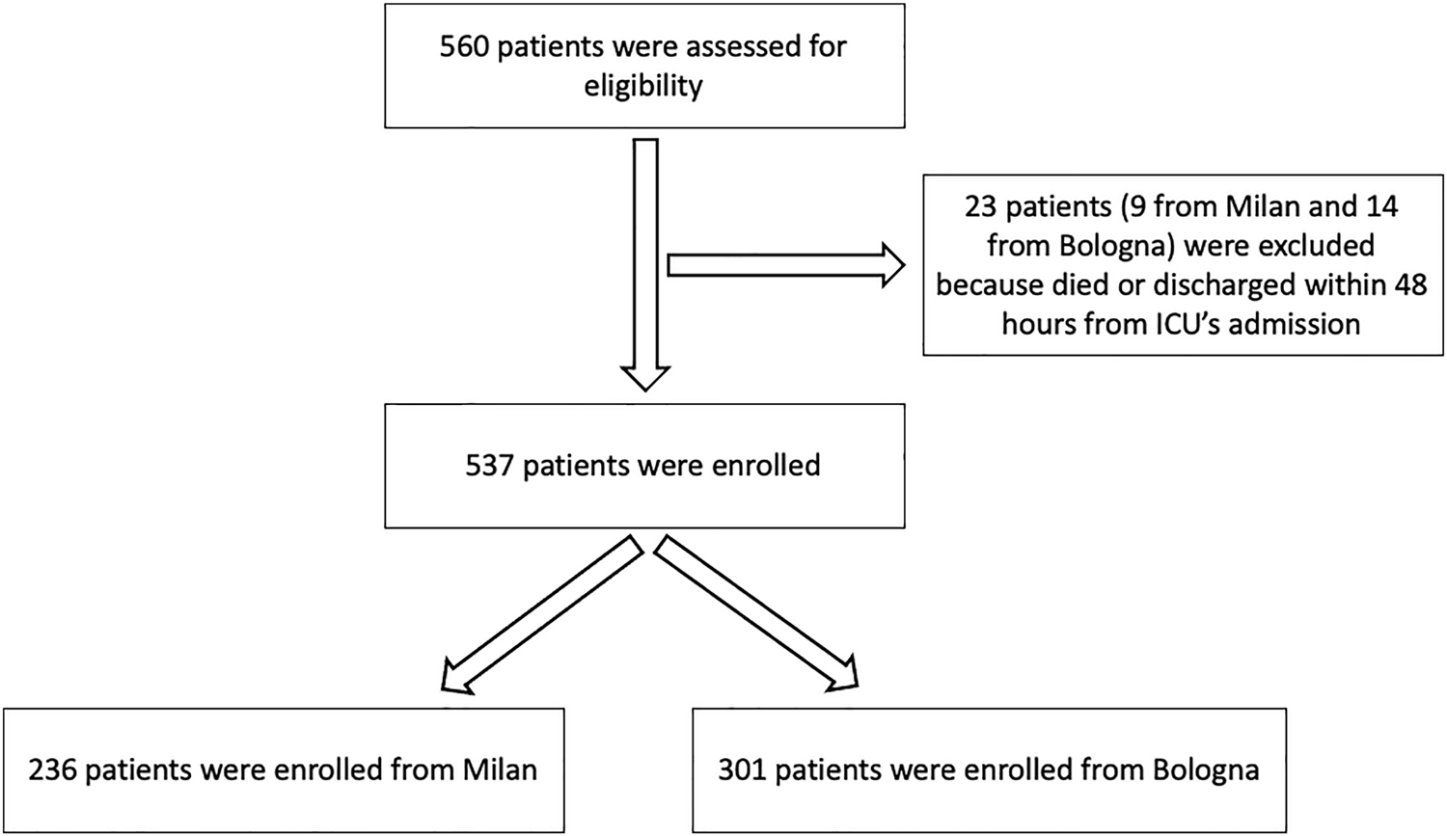
Eligibility process

Out of 560 patients assessed for eligibility (Fig. 1), we excluded 23 patients because died or discharged within 48 h from ICU’s admission (9 patients from Milan and 14 patients from Bologna). Five hundred-thirty-seven critically ill COVID-19 patients (74.9% male; median age 65 years, IQR 57–72) were admitted in ICU for at least 48 h during the study period (Table 1). At the time of admission, their median sequential organ failure assessment (SOFA) score was 9 (IQR 7–11). The median length of ICU stay was 13 days (IQR 7.5–22); 85.3% of patients required intubation and 258 (48%) died.

**Table 1:** Comparison of ICU admitted COVID-19 patients with and without BSI

Variables	Total *N* = 537 (%)	No-BSI *N* = 272 (%)	BSI *N* = 265 (%)	*p*
Demographic data				
Age (years) (median, IQR)	65 (57–72)	65 (56–72)	65.8 (58–72)	0.78
Sex, male	402 (74.9)	198 (72.8)	204 (77)	0.28
Underlying diseases				
Obesity	184 (34.3)	99 (36.4)	85 (32.1)	0.32
Diabetes	90 (16.8)	41 (15.1)	49 (18.5)	0.3
Lung diseases	69 (12.8)	35 (12.9)	34 (12.8)	1
Heart diseases	105 (19.6)	56 (20.6)	49 (18.5)	0.59
Metabolic disorders	41 (7.6)	23 (8.5)	18 (6.8)	0.52
Liver diseases	19 (3.5)	11 (4)	8 (3)	0.64
Renal diseases	52 (9.7)	24 (8.8)	28 (10.6)	0.56
Oncological diseases	40 (7.4)	25 (9.2)	15 (5.7)	0.14
Immunosuppression	27 (5)	11 (4)	16 (6)	0.33
Charlson index score (median, IQR)	3 (2–4)	3 (1–4)	3 (2–4)	0.59
COVID-19				
Days from symptoms onset to hospital admission (median, IQR)	7 (4–9)	7 (5–9)	7 (4–9)	0.22
Days from hospital admission to ICU admission (median, IQR)	2 (0–5)	2 (0–5)	3 (1–5)	0.38
SOFA score at BSI onset (median, IQR)	9 (7–11)	9 (7–10)	10 (8–12)	< 0.001
IOT	458 (85.3)	204 (75)	254 (95.8)	< 0.001
Length of IOT (days) (median, IQR)	12 (7–20)	8 (5–14)	16 (9–25)	< 0.001
Days from ICU admission to IOT (median, IQR)	0 (0–1)	0 (0–1)	0 (0–1)	0.7
Center		189 (69.5)	112 (42.3)	< 0.001
Treatments				
Tocilizumab	133 (48.9)	133 (48.9)	95 (35.8)	0.002
Remdesivir	45 (16.5)	45 (16.5)	57 (21.5)	0.15
Heparin	220 (80.9)	220 (80.9)	206 (77.7)	0.4
Steroids	181 (66.5)	181 (66.5)	185 (69.8)	0.46
Antibiotic treatment within 30 days from ICU’s admission	235 (86.4)	235 (86.4)	252 (95.1)	0.001
Outcome				
Length of ICU stay (days) (median, IQR)	9 (5–15)	9 (5–15)	18 (12–31.5)	< 0.001
All cause 30-day mortality	115 (42.3)	115 (42.3)	143 (54)	0.007
Days from ICU admission to death (median, IQR)	10 (6–14)	10 (6–14)	16 (10–23)	< 0.001

During the ICU stay, in 265 patients (49.3%) at least one episode of bacteremia (64.8% in Milan and 37.2% in Bologna centers) was diagnosed. The crude incidence rate of bacteremia in Milan and Bologna was respectively 92/1000 patients days (95% CI 79–108) and 27/1000 patients days (95% CI 23 – 33).

To interpret these findings, it is necessary to consider the discrepancy in the total number of blood cultures performed between the two centers. In fact, in a random sample of 10% of patients matched by demographic and clinical features, the number of blood cultures performed divided per day of ICU stay in Milano is three times higher than Bologna: 0.45 vs 0.15.

As shown in Table 2 the source of BSI was unknown in 106 cases (40%), central line-associated BSI in 87 cases (32.9%), ventilator-associated pneumonia in 41 cases (15.5%), urinary tract infection in 27 cases (10.2%), intra-abdominal infections in four cases (1.5%).

**Table 2:** Source of infection

Source of infection	1st wave	2nd wave	Total
	*n* (%)	*n* (%)	*n* (%)
Primary	48 (32.2)	58 (50)	106 (40)
CLABSI	56 (37.6)	31 (26.7)	87 (32.9)
LUNG	29 (19.5)	12 (10.3)	41 (15.5)
UTI	15 (10.1)	12 (10.3)	27 (10.2)
IAI	1 (0.7)	3 (2.6)	4 (1.5)
Total	149	116	265

*CLABSI* central line associated bloodstream infection, *UTI* urinary tract infection, *IAI* intra-abdominal infection

305 isolates were identified (Table 3). The majority (208, 68.2%) were Gram-positive bacteria, prevalently *Enterococcus* spp. (97, 31.8%), followed by coagulase-negative staphylococci (43, 14.1%) and *Staphylococcus aureus* (30, 9.8%). Gram-negative bacteria resulted in 28.9% of the isolates, and *Candida* spp. in 3%. Fifty-five isolates were MDR including: *Staphylococcus aureus* methicillin-resistant (*n* = 16, 5.2%), *Enterobacteriaceae* extended-spectrum beta-lactamase (*n* = 10, 3.3%), Carbapenem resistance *Acinetobacter baumannii* (*n* = 10, 3.3%), multi-drug resistance *Pseudomonas aeruginosa* (*n* = 9, 3%), carbapenemase producing *Enterobacteriaceae* (*n* = 6, 2%); vancomycin resistance enterococci (*n* = 4, 1.3%).

**Table 3:** Type of isolates

Type of isolates	1st wave	2nd wave	Total
	*n* (%)	*n* (%)	*n* (%)
Total	165 (100)	140 (100)	305 (100)
Gram +	107 (64.8)	101 (72.1)	208 (68.2)
*Enterocuccus* spp.	50 (30.3)	47 (33.6)	97 (31.8)
VRE	2 (1.2)	2 (1.4)	4 (1.3)
*S. aureus*	12 (7.3)	18 (12.9)	30 (9.8)
MRSA	10 (6.1)	6 (4.3)	16 (5.2)
CoNS	41 (24.8)	2 (1.4)	43 (14.1)
Viridans *Streptococci*	1 (0.6)	6 (4.3)	7 (2.3)
Other	3 (1.8)	3 (2.1)	6 (2)
Gram -	52 (31.5)	36 (25.7)	88 (28.9)
*Enterobacteriaceae*	25 (15.2)	19 (13.6)	44 (14.4)
ESBL	9 (5.5)	1 (0.7)	10 (3.3)
CPE	6 (3.6)	0 (0)	6 (2)
*Enterobacter* spp	4 (2.4)	11 (7.9)	15 (4.9)
*Pseudomonas aeruginosa*	14 (8.5)	5 (3.6)	19 (6.2)
MDR	7 (4.2)	2 (1.4)	9 (3)
*S. maltophilia*	0 (0)	0 (0)	0 (0)
*A. baumannii*	9 (5.5)	1 (0.7)	10 (3.3)
CRAB	9 (5.5)	1 (0.7)	10 (3.3)
Yeasts	6 (3.6)	3 (2.1)	9 (3)
*Candida albicans*	4 (2.4)	2 (1.4)	6 (2)
*Candida parapsilosis*	2 (1.2)	1 (0.7)	3 (1)

### Risk factors associated with the development of bacteremia

As shown in Table 1, patients who developed BSI had a higher median SOFA score upon admission (10, IQR 8–12 *vs* 9, IQR 7–10; *p* < 0.001), required more frequently mechanical ventilation (95.8% *vs* 75%; *p* < 0.001), had a longer duration of mechanical ventilation (16, IQR 9–25 *vs* 8, IQR 5–14; *p* < 0.001), and prior antibiotic exposure was more frequent (95.1% *vs* 86.4%; *p* 0.001) among BSI than non-BSI patients. The probability of receiving antibiotic therapy increased with the days spent in intensive care. Of patients stayed less than 7 days in ICU about 50% received antimicrobial treatment, 65% of whom who stayed between 7 and 10 days and 81% of patients who stayed for more than ten days. Significant differences were also observed for all cause 30-day mortality (54% *vs* 42.3%; *p *0.007), and length of ICU stay (18, IQR 12–31.5 *vs* 9, IQR 5–15). None of the patients included into the study received ECMO.

Univariable and multivariable analyses of factors potentially associated with the occurrence of BSI are shown in Supplementary Materials 1–2. In univariable analysis epidemic wave 2 *vs* 1 [unadjusted cause-specific HR 1.71 (95% CI 1.33–2.20)], the center hospital: Bologna *vs* Milano [csHR 0.22 ( 95% CI 0.17–0.29)], SOFA score [(csHR 1.07 (95% CI 1.02–1.12)], treatment with remdesivir [csHR 1.38 ( 95% CI 1.02–1.85)], treatment with tocilizumab [csHR 0.45 (95% CI 0.34–0.58)] and mechanical ventilation [csHR 1.68 (95% CI 1.03–2.72)] are associated with the development of BSI. In multivariate analysis adjusted for center (Bologna vs Milan), wave, sex, age, obesity, days from symptoms to ICU’s admission, Charlson and SOFA Scores, tocilizumab, remdesivir, steroids, orotracheal intubation (IOT), while considering tocilizumab and IOT as time-dependent variables, only SOFA score [csHR 1.07 (95% CI 1.02–1.11)], Charlson score [csHR 1.16 (95% CI 1.07–1.27)] and center hospital: Bologna *vs* Milano [csHR 0.22 (95% CI 0.15–0.31)] showed an independent association with the BSI. No association between immunosuppressive therapy as steroids and tocilizumab and bacteremia has emerged. In the analysis restricted per center, although statistical significance was not always reached, the risk factors associated with bacteremia remained consistent in the two centers (Supplementary Materials 3). According to the VIF values, no multicollinearity was found among the independent variables included.

## Discussion

To the best of our knowledge regarding other studies investigating the risk factors associated with bacteremia this is the one with the highest number of patients recruited. Moreover, compared to most of the similar studies currently available, it includes the period of two-wave pandemic and is not limited only to the first.

We found that previous comorbidities and severity of clinical conditions at ICU admission are risk factors for developing bacteremia, while immunosuppressive therapy does not represent a risk factor for this event.

In our multicenter study, we reported an incidence rate of 92/1000 patients days in Milan and 27/1000 patients days in Bologna centers both of which are higher than 5–19 episodes per 1000 patient-days registered in other studies valuing non-COVID-19 populations [7, 14, 15]. The difference between these two centers and the association confirmed through multivariable analysis of the BSI with the center can be partially explained by the different number of blood cultures performed and reported in the results. The considerable disproportion of blood cultures can be explained by a different number of positive blood cultures mainly due to coagulase-negative *Staphylococci* or other contaminant agents. Moreover, in critically ill patients, the clinical approach to obtain blood cultures can widely change between different centers. Some clinicians prefer to perform surveillance cultures to better determine patients’ bacteria colonization to then be able to choose the right antibiotic therapy in case of an infectious event; others carry out cultural examinations only in the presence of a clinically relevant event to avoid the collection of misleading information. Consequently, it could not be excluded that the difference observed in the incidence between the two centers could be driven by a different decision making before blood culture request rather than a true difference in the incidence of BSI. In the COVID-19 era, as Sepulveda et al. pointed out, even in totally different countries and settings, like in New York’s hospital a high number of blood cultures was performed leading to a high prevalence of bacteremia due to germs like CoNS, difficult to be clinically interpreted [16].

Various hypotheses can be put forth to explain this high incidence. Many studies have described the presence of an immune system dysregulation in COVID-19 patients [17], but to date there are no studies that document a real association with this evidence and the development of super-infections such as BSI. Furthermore, the sole admission to ICU is itself a risk factor for the development of healthcare-related infections. In the COVID-19 era, infection control measures inevitably failed as the need to deal with an unprecedented health emergency arose [5]. Although the role of extensive use of immunosuppressants such as steroids, anti-IL1 and above all tocilizumab, is still debated, it could be speculated that it may have contributed to alter the balance of immune defenses by favoring the development of super-infections [18]. Some available evidence in literature, like the work published by Giacobbe et al., which includes all ICU admitted patients, seems to consider steroids and tocilizumab use as a factor associated to an increased risk of BSI development [10]. In our study, no association between steroids or tocilizumab therapy and development of bacteremia has been observed. This is in line with the report by Abelenda-Alonso et al. who performed a severity matched case–control study in a large multicenter prospective cohort of hospitalized adults with COVID-19 where 100 patients presented 142 episodes of BSI, mainly caused by Coagulase-negative *Staphylococci*, *Enterococcus faecalis* and *Pseudomonas aeruginosa*. In the multivariate analysis, the use of these immunomodulatory drugs was not associated with an increased risk of BSI [9]. Conversely, in the paper published by Giacobbe et al. in which all patients recruited in the study were admitted in the ICU, steroid and tocilizumab seem to increase the risk of developing BSIs [10].

The analysis of risk factors related to the development of BSI performed on our cohort of patients is consistent with what was described so far in literature [19]. The burden of comorbidities assessed by Charlson score and the disease severity upon ICU admission assessed by SOFA score were found to be independently associated to BSI occurrence.

Regarding the most frequently isolated pathogens, despite the epidemiological difference linked to two different centers, the prevalence of the strains in Milan and Bologna was similar. Two-thirds of the pathogens were gram-positive, with the majority represented by Enterococci. Many studies, conducted during the first wave of the Italian epidemic, showed a high incidence of Enterococci in COVID-19 critically ill patients [20]. Other studies tried to demonstrate the possible origin of Enterococci bacteremia using Whole Genome Sequencing (WGS) technique suggesting that this frequent event seems not related to an hospital outbreak [21].

To answer this question Gaibani et al. analyzed the intestinal microbiome of patients with COVID-19 compared with healthy subjects with similar characteristics and noted that the microbiome of COVID-19 subjects was much richer in *Enterococcus* than the control group. This feature was also overrepresented in patients developing BSIs and admitted to the ICU [22]. Regarding the MDR pathogens which may cause bacteremia, it is important to note that the pandemic itself has changed the microbiological environment responsible in the colonization by MDR germs in patients admitted to the ICU as highlighted by Pascale et al. In this study the CR-Ab colonization and CR-Ab infection increased by 7.5 and 5.5 times, respectively, during the first wave of Sars-CoV-2 infection compared to the same period of the previous year [23]. It is also notable how although hospitals attempted to maintain the best infection control practices, the COVID-19 pandemic has presented some unique challenges, such as continuously changing recommendations, patient surges and resource shortages leading to an inevitable increase in the rate of healthcare-related infections [24].

Our study has several limitations. First, it is a retrospective study. Second, due to its multicentric nature, we must consider the differences that occur in the geographical distance of the two centers. In particular, the study was conducted during the first two waves of the Italian epidemic when there were no guidelines yet that could help clinicians in their decisions thus bringing to very different kinds of therapeutic interventions being carried out. Furthermore, centers were also differently concerned about epidemiological factors resulting in a possible alteration of microbiological isolates. In the end, a sampling bias related to a different threshold for blood cultures request could explain the difference in prevalence of BSI observed in Milan and Bologna centers.

## Conclusions

In our multicenter study conducted on patients hospitalized for COVID-19 in intensive care, a high incidence of BSI was observed. A high SOFA score and a high Charlson score resulted associated with BSI’s development. Conversely, immunosuppressive therapy like steroids and tocilizumab, has no role in increasing the risk of bacteremia.

## Supplementary Information

Below is the link to the electronic supplementary material.

Supplementary file1 (DOCX 16 KB)

Supplementary file2 (DOCX 13 KB)

Supplementary file3 (DOCX 19 KB)
