# Supplementary material for: Risk factors associated with bacteremia in COVID-19 patients admitted to intensive care unit: a retrospective multicenter cohort study
Source: Infection. 2022 Jun 10;51(1):129–36. doi: 10.1007/s15010-022-01853-4 (PMC9185127; doi:10.1007/s15010-022-01853-4)
Supplement: Supplementary file 1 — Supplementary file1 (DOCX 16 KB) [file 15010_2022_1853_MOESM1_ESM.docx]

**Supplemental Materials: Univariable analysis**

| Variables | p-value | Adjusted cause-specific HR (95% CI) |
| --- | --- | --- |
| Wave: 2 vs. 1 | **<.0001** | **1,71 (1,33 – 2,2)** |
| Hospital: BO vs. MI | **<.0001** | **0,22 (0,17 – 0,29)** |
| Sex: Female vs. Male | 0,559 | 0,92 (0,69 – 1,22) |
| Age (x 1 year more) | 0,379 | 0,99 (0,98 – 1,01) |
| Obesity: Yes vs. No | 0,472 | 0,91 (0,70 – 1,18) |
| Days from symptoms to ICU (x 1 day more) | 0,464 | 1,01 (0,99 – 1,03) |
| Charlson Score (x 1 unit more) | 0,083 | 1,07 (0,99 – 1,16) |
| Tocilizumab: Yes vs. No | **<.0001** | **0,46 (0,34 – 0,58)** |
| Remdesivir: Yes vs. No | **0,035** | **1,38 (1,02 – 1,85)** |
| Steroids: Yes vs. No | 0,863 | 1,02 (0,79 – 1,33) |
| Sofa Score (x 1 unit more) | **0,003** | **1,07 (1,02 – 1,12)** |
| IOT: Yes vs. No | **0,036** | **1,68 (1,04 – 2,72)** |
