# Supplementary material for: Risk factors associated with bacteremia in COVID-19 patients admitted to intensive care unit: a retrospective multicenter cohort study
Source: Infection. 2022 Jun 10;51(1):129–36. doi: 10.1007/s15010-022-01853-4 (PMC9185127; doi:10.1007/s15010-022-01853-4)
Supplement: Supplementary file 2 — Supplementary file2 (DOCX 13 KB) [file 15010_2022_1853_MOESM2_ESM.docx]

**Supplemental Materials: Multivariable analysis**

| Variables | p-value | Adjusted cause-specific HR (95% CI) |
| --- | --- | --- |
| Wave: 2 vs. 1 | 0,958 | 0,99 (0,69-1,42) |
| Hospital: BO vs. MI | **<.0001** | 0,22 (0,16-0,31) |
| Sex: Female vs. Male | 0,894 | 0,98 (0,73-1,32) |
| Age (x 1 year more) | 0,407 | 0,99 (0,98-1,01) |
| Obesity: Yes vs. No | 0,982 | 1,00 (0,77-1,31) |
| Days from symptoms to ICU (x 1 day more) | 0,720 | 0,99 (0.98-1,02) |
| Charlson Score (x 1 unit more) | **<.001** | 1,16 (1,07-1,26) |
| Tocilizumab: Yes vs. No | 0,245 | 0,82 (0,59-1,14) |
| Remdesivir: Yes vs. No | 0,727 | 1,06 (0,77-1,47) |
| Steroids: Yes vs. No | 0,268 | 1,22 (0,86-1,74) |
| Sofa Score (x 1 unit more) | **0,004** | 1,07 (1,02-1,12) |
| IOT: Yes vs. No | 0,051 | 1,67 (0,99-2,78) |
