# Supplementary material for: Risk factors associated with bacteremia in COVID-19 patients admitted to intensive care unit: a retrospective multicenter cohort study
Source: Infection. 2022 Jun 10;51(1):129–36. doi: 10.1007/s15010-022-01853-4 (PMC9185127; doi:10.1007/s15010-022-01853-4)
Supplement: Supplementary file 3 — Supplementary file3 (DOCX 19 KB) [file 15010_2022_1853_MOESM3_ESM.docx]

**Supplementary material:** Analysis restricted per center.

| **Milano** | *Variables* | *p-value* | *Unadjusted cause-specific HR (95% CI)* | | | *p-value* | *Adjusted cause-specific HR (95% CI)* | | |
| --- | --- | --- | --- | --- | --- | --- | --- | --- | --- |
|  | Wave: 2 vs. 1 | 0,117 | 1,298 | 0,937 | 1,799 | 0,461 | 1,293 | 0,653 | 2,561 |
|  | Sex: Female vs. Male | 0,312 | 0,818 | 0,553 | 1,208 | 0,271 | 0,793 | 0,524 | 1,199 |
|  | Age (x 1 year more) | 0,895 | 0,999 | 0,985 | 1,013 | 0,340 | 0,993 | 0,978 | 1,008 |
|  | Obesity: Yes vs. No | 0,480 | 1,132 | 0,802 | 1,599 | 0,781 | 1,052 | 0,735 | 1,507 |
|  | Days from symptoms to RIA (x 1 day more) | 0,268 | 0,987 | 0,964 | 1,010 | 0,731 | 0,996 | 0,971 | 1,021 |
|  | Charlson Score Unadjusted for age (x 1 unit more) | 0,075 | 1,109 | 0,990 | 1,243 | 0,116 | 1,103 | 0,976 | 1,245 |
|  | Tocilizumab: Yes vs. No | 0,326 | 0,796 | 0,506 | 1,255 | 0,852 | 0,948 | 0,542 | 1,657 |
|  | Remdesivir: Yes vs. No | 0,603 | 0,911 | 0,640 | 1,296 | 0,808 | 0,953 | 0,648 | 1,403 |
|  | Steroids: Yes vs. No | 0,260 | 1,213 | 0,867 | 1,696 | 0,993 | 0,997 | 0,529 | 1,880 |
|  | Sofa Score (x 1 unit more) | 0,481 | 1,020 | 0,966 | 1,076 | 0,634 | 1,015 | 0,955 | 1,079 |
|  | IOT: Yes vs. No | **0,017** | **2,272** | 1,155 | 4,471 | **0,032** | **2,174** | 1,071 | 4,415 |
|  |  |  |  |  |  |  |  |  |  |
| **Bologna** | *Variables* | *p-value* | *Unadjusted cause-specific HR (95% CI)* | | | *p-value* | *Adjusted cause-specific HR (95% CI)* | | |
|  | Wave: 2 vs. 1 | 0,944 | 1,017 | 0,639 | 1,617 | 0,333 | 0,771 | 0,456 | 1,305 |
|  | Sex: Female vs. Male | 0,668 | 1,097 | 0,718 | 1,677 | 0,887 | 1,033 | 0,662 | 1,611 |
|  | Age (x 1 year more) | 0,358 | 1,009 | 0,990 | 1,030 | 0,864 | 0,998 | 0,975 | 1,021 |
|  | Obesity: Yes vs. No | 0,692 | 0,924 | 0,623 | 1,369 | 0,884 | 1,031 | 0,683 | 1,557 |
|  | Days from symptoms to RIA (x 1 day more) | 0,721 | 1,006 | 0,973 | 1,041 | 0,208 | 1,021 | 0,988 | 1,056 |
|  | Charlson Score Unadjusted for age (x 1 unit more) | **0,001** | **1,224** | 1,090 | 1,375 | **0,014** | **1,166** | 1,031 | 1,319 |
|  | Tocilizumab: Yes vs. No | 0,078 | 0,701 | 0,473 | 1,040 | 0,084 | 0,686 | 0,447 | 1,052 |
|  | Remdesivir: Yes vs. No | 0,939 | 1,023 | 0,572 | 1,829 | 0,682 | 1,144 | 0,601 | 2,178 |
|  | Steroids: Yes vs. No | 0,551 | 1,139 | 0,742 | 1,750 | 0,913 | 1,027 | 0,635 | 1,663 |
|  | Sofa Score (x 1 unit more) | **<.0001** | **1,182** | 1,099 | 1,272 | **<.0001** | **1,173** | 1,084 | 1,268 |
|  | IOT: Yes vs. No | 0,814 | 1,087 | 0,542 | 2,180 | 0,688 | 1,168 | 0,548 | 2,486 |
